# A reconstruction of a medical history from administrative data: with an application to the cost of skin cancer

**DOI:** 10.1186/s13561-015-0042-x

**Published:** 2015-02-11

**Authors:** David Rowell, Louisa G Gordon, Catherine M Olsen, David C Whiteman

**Affiliations:** 1 Centre for Applied Health Economics, Griffith Health Institute, Griffith University, Logan Campus, University Drive, Meadowbrook, Brisbane 4131 Australia; 2 The University of Queensland, UQ Centre for Clinical Research – Asia-Pacific Centre for Neuromodulation, Building 71/918, Herston, Brisbane 4029 Australia; 3 QIMR Berghofer Medical Research Institute, Population Health Department, Royal Brisbane Hospital, Herston, Brisbane 4029 Australia; 4 NHMRC Centre for Research Excellence in Sun and Health, Queensland University of Technology, Kelvin Grove, Brisbane 4059 Australia

**Keywords:** C10, I11, Keratinocytic cancer, Cost of illness, Administrative data

## Abstract

The medical record is a repository of clinical data, which can greatly enhance the quality of health and healthcare analysis. Administrative data are collected for the purpose of billing and reimbursement, and are valued by health researchers because the data are routinely audited to maintain accurate financial records. However, the quantity of incorporated clinical data can be variable. In this paper we reconstruct the medical record from health service invoices to estimate the cost of treating keratinocytic cancer (KC). The data from an epidemiological survey were linked to an administrative data set supplied by the national health insurer. A matched sampling technique with multivariable analysis was used to estimate cost. A KC treatment was identified with 42 service codes which explicitly nominated treatment of a KC. Algorithms identifying comorbities potentially correlated with KC were constructed from the service codes. The annual cost of a KC treatment was estimated to be AU$667 per individual. The average cost of explicit KC treatments was AU$231, while the cost of generic procedures used to treat KC was AU$436. Our ability to accurately control for the medical history enabled our analysis to quantify and describe the constituent costs of KC treatment.

**Electronic supplementary material:**

The online version of this article (doi:10.1186/s13561-015-0042-x) contains supplementary material, which is available to authorized users.

## Background

Empirical analysis in healthcare can be enhanced by controlling for the confounding effects of comorbidities documented within a patient’s medical record. A patient’s medical history can be obtained by direct interview or interrogation of their medical record. However, self-reported medical histories can be subject to a reporting bias, while review of the medical record may be unfeasible or costly. There is a growing appreciation of the benefits of using administrative data to conduct health research [1-3]. Many health insurers, both public and private, generate large datasets for the purpose of either reimbursing physicians or invoicing patients. Although not designed for research, these administrative data, which include item codes identifying discrete episodes of care, are a potentially rich source of clinical information.

In this paper, our aim is to reconstruct a patient’s medical history from the service codes contained within an administrative dataset, to facilitate the estimation of the cost of treating the non-melanoma skin cancers (and which are more accurately described as keratinocyte cancers (KC)). Keratinocyte cancers, which comprise both basal cell carcinomas (BCC) and squamous cell carcinoma (SCC), are cancers with high incidence but low mortality. Worldwide, KC are the most prevalent cancers affecting white-skinned individuals and their incidence is rising rapidly in many countries [4]. High reported incidence rates of KC in Australia (1,170 per 100,000) [5] and the United States (233 per 100,000) [6] ensure that these cancers remain the most costly and fifth most costly to treat in Australia [7] and the United States [8], respectively. However, due to their low mortality rate, many national cancer registries have incomplete or non-existent reporting of KC [4,9]. Therefore, the analysis of administrative data may be particularly useful for health service research of diseases such as KC, where conventional data sources may otherwise be incomplete.

Our literature review identified nine studies that estimated the cost of treating KC using administrative data [7,8,10-16]. Although each study analysed a different dataset, their methods were broadly similar. Typically, they identified an episode of treatment for KC within their data. They then ascribed a mean cost per KC episode before reporting an aggregate cost, for their jurisdiction of interest. However, there was significant heterogeneity with respect to how KC treatments were defined and costed. For example Souza et al. [17] relied on “expert opinion” to define a KC treatment. Data from the Brazilian National Health Service and medical costs supplied by the Brazilian Medical Association were used to estimate aggregate costs. An Australian study published by Fransen et al. analysed an administrative dataset obtained from the national health insurer, Medicare Australia [7]. These data included a unique item code for each medical service delivered. A treatment for KC was identified if one of 37 item codes, which denoted excision of a BCC or SCC, were identified within the data. The corresponding costs were summed and reported. However, the costs of ancillary services such as histology or pharmaceuticals were not included nor were the costs of attendance fees.

The surveyed literature almost entirely reports average and aggregate costs. Three European studies [10-12] identified a KC using the International Classification of Diseases (ICD) codes and costs of treatment were estimated by using national Diagnostic Related Group (DRG) cost weights. Outpatient costs were included in an *ad hoc* manner, using sub-samples of outpatient cost data. The three US studies [8,14,18] used data collected for the Medicare Current Beneficiary Survey (MCBS) 1992–95 to derive cost estimates for KC. Data from each Medicare claim was linked to the appropriate specialty [14] and costs summed and reported. While these costing methods were no doubt sound, simply reporting an aggregate cost provides little opportunity for researchers and policy makers to further integrate these estimates. As the focus of these studies tended to be on the procedure rather than the individual questions concerning who is consuming which KC treatments remained largely unanswered.

We could identify one study, which employed a different method. Bentzen et al. [16] estimate the cost of treating KC in Denmark. The Danish National Patient Register tracks all inpatient and outpatient health costs. A KC was identified using ICD codes. All individuals treated for a KC in the period 2004 to 2008 were matched to a set of controls (at a ratio of 1:4) on four criteria (age, sex, civil status and residence). The costs of treating KC were calculated as the average annual excess costs per year for patients after diagnosis relative to the matched control cohort. The principal strength of the paper by Bentzen et al. [16] lay in its capacity to analyse patient records, which linked cost and demographic data. While KC incidence was identified by ICD code, the cost of a treatment was not predetermined. Instead Bentzen et al. [16] estimated the cost of treating KC conditional upon a set of demographic controls. An advantage of this approach is that a description of treatment costs can be developed. The principal limitation was that Bentzen et al. [16] only controlled for age, sex, civil status and residence. Human disease can be correlated for a variety of genetic, environmental and social reasons. If available, controls for medical history may have been beneficial.

In this paper, we derive a set of dichotomous variables from treatments documented in an administrative dataset to capture the medical history. Our aims were twofold. Firstly, we control for cost of treating comorbid disease to report an estimate the cost of KC treatment. Secondly, we identified and classified the medical treatments utilised by patients with KC. Controlling for medical history can not only result in more accurate measures of cost but also offer a deeper understanding of component costs.

## Methods

### Data

In 2011, the QSkin study enrolled 43,794 individuals aged 40 to 69 years selected at random from the Queensland electoral roll [19,20]. Overall, 46% of the respondents were male with a mean age of 56 years [19]. The respondents reported their level of sun exposure, skin phenotype, history of skin cancer, demographic and socio-economic characteristics [19]. Ethical approval for the study was received from the QIMR Berghofer Institute of Medical Research Human Research Ethics Committee and the Department of Health. Consent was obtained to link survey data supplied by the respondent to individual level cost data obtained from two publically funded health programs administered by Medicare Australia, the Pharmaceutical Benefits Scheme (PBS) and the Medical Benefits Scheme (MBS). The PBS subsidises the cost of approved pharmaceuticals. The MBS subsidises (i) fee-for-service medical care provided by GPs and specialist physicians delivered in their private consulting rooms and (ii) medical care provided to private patients treated in private and public hospitals. However, the MBS excludes medical services provided to public inpatients. Other inpatient costs (private and public) not included in this cost analysis are non-medical services (nursing, allied health and ancillary services), hospital consumables, administrative overheads and capital depreciation.

Thus, the proportion of the total KC treatment costs captured by this sub-set of healthcare costs identifiable by a MBS item number is uncertain. A national survey of individuals treated for KC (n = 2502) in 2002 reported that 51.1% respondents were treated by general practitioners, 17.6% by dermatologists, 10.3% in skin cancer clinics, 5.9% by plastic surgeons, 3.4% other surgeons and 1% other and 9.2% not stated [21]. However, only 1.6% of respondents indicated that their last KC treatment was conducted in hospital [21]. While these categories are (i) not necessarily mutually exclusive (e.g., plastic surgery can be conducted in a hospital), and (ii) report treatments not costs, it is likely that medical treatments denoted by an MBS item number comprise a very high proportion of total KC treatment costs.

A sub-sample comprised of 2,000 randomly selected individuals with KC matched 1:1 on gender and 5-year age categories, to a group of controls. KC was identified by 42 MBS codes^a^, which unequivocally indicated a treatment for a BCC or SCC (see Table 1). Data cleaning identified inconsistencies in 0.4% of the cases and 11.95% of the controls, which were subsequently removed. The final sample of 3,753 individuals was comprised of 1,992 cases with KC and 1,761 controls. After matching the case and control cohorts contained an equal proportion of males and females. Individuals with KC were slightly older (57.2 years versus 55.7 years) more likely to be white (95.8% versus 92.3%), born in Australia (85.2% versus 79.6%), less likely to be employed full-time (39.8% versus 45.5%) and not have private health insurance (73.6% versus 67.6%).

**Table 1:** **Observed category 1 costs**

**Medical benefits scheme item description**	**MBS code**	**Freq.**	**Mean (AU$)**	**Total (AU$)**
Diagnostic biopsy of skin or mucous membrane, specimen sent for biopsy	30071	1956	49	96,351
Removal of malignant neoplasm of skin by serial curettage or carbon dioxide laser excision-ablation: < 10 lesions	30196	433	96	41,590
Removal of malignant neoplasm of skin by serial curettage or carbon dioxide laser excision-ablation: > 10 lesions	30197	8	680	5,438
Removal of malignant neoplasm of skin by cryotherapy: <10 lesions	30202	121	42	5,082
Removal of malignant neoplasm of skin by cryotherapy: > 10 lesions	30203	16	145	2,328
Removal of malignant neoplasm of skin and cartilage by cryotherapy: > 10 lesions	30205	0	0	0
Mircographically controlled serial excision of skin tumour with histological examination: < 6 lesions	31000	15	826	12,383
Mircographically controlled serial excision of skin tumour with histological examination: 7–12 lesions	31001	6	1,111	6,667
Mircographically controlled serial excision of skin tumour with histological examination: > 13 lesions	31002	2	1,225	2,451
*Removal from nose, eyelid, lip, ear, digit or genitalia by surgical excision*				
Removal of BCC or SCC with malignancy confirmed: < 10 mm diameter	31255	169	183	30,950
Removal of residual BCC or SCC by original GP, specimen sent to histology: Original tumour < 10 mm diameter	31256	15	165	2,475
Removal of residual BCC or SCC by non-original GP, specimen sent to histology: Original tumour < 10 mm diameter	31257	3	264	791
Removal of recurrent BCC or SCC, malignancy confirmed by histology: Original tumour < 10 mm diameter	31258	1	185	185
Removal of BCC or SCC with malignancy confirmed: >10 mm diameter	31260	46	218	10,038
Removal of residual BCC or SCC by original GP, specimen sent to histology: Original tumour >10 mm diameter	31261	6	291	1,746
Removal of residual BCC or SCC by non-original GP, specimen sent to histology: Original tumour > 10 mm diameter	31262	1	422	422
Removal of recurrent BCC or SCC, malignancy confirmed by histology: Original tumour > 10 mm diameter	31263	0	0	0
*Removal from face, neck (anterior to the sternomastoid muscles) or lower leg (mid-calf to ankle) by surgical excision*				
Removal of BCC or SCC, malignancy confirmed by histology, <10 mm diameter	31265	338	165	55,710
Removal of residual BCC or SCC, by original GP, specimen sent to histology: Original tumour <10 mm diameter	31266	12	177	2,126
Removal of residual BCC or SCC, by non-original GP, specimen sent to histology: Original tumour <10 mm diameter	31267	1	154	154
Removal of recurrent BCC or SCC, malignancy confirmed by histology: Original tumour >10 mm diameter	31268	1	154	154
Removal of BCC or SCC with malignancy confirmed: 10-20 mm diameter	31270	128	213	27,276
Removal of residual BCC or SCC by original GP, specimen sent to histology: Original tumour 10-20 mm diameter	31271	1	106	106
Removal of residual BCC or SCC by non-original GP, specimen sent to histology: Original tumour 10-20 mm diameter	31272	0	0	0
Removal of recurrent, BCC or SCC, malignancy confirmed: Original tumour 10-20 mm diameter	31273	0	0	0
Removal of BCC or SCC, malignancy confirmed: >20 mm diameter	31275	25	246	6,158
Removal of residual BCC or SCC, by original GP, specimen sent to histology: Original tumour >20 mm diameter	31276	2	188	376
Removal of residual BCC or SCC, by non-original GP; Original tumour >20 mm diameter	31277	1	101	101
Removal of recurrent BCC or SCC, malignancy confirmed: Original tumour > 20 mm diameter	31278	0	0	0
*Removal from other body areas by surgical excision*				
Removal of BCC or SCC, malignancy confirmed by histology, <10 mm diameter	31280	620	144	89,155
Removal of residual BCC or SCC, by original GP, specimen sent to histology: Original tumour <10 mm diameter	31281	7	171	1,197
Removal of residual BCC or SCC, by non-original GP, specimen sent to histology: Original tumour <10 mm diameter	31282	2	141	282
Removal of recurrent BCC or SCC, malignancy confirmed by histology: Original tumour >10 mm diameter	31283	1	131	131
Removal of BCC or SCC with malignancy confirmed: 10-20 mm diameter	31285	267	179	47,706
Removal of residual BCC or SCC by original GP, specimen sent to histology: Original tumour 10-20 mm diameter	31286	2	133	267
Removal of residual BCC or SCC by non-original GP, specimen sent to histology: Original tumour 10-20 mm diameter	31287	2	178	355
Removal of recurrent, BCC or SCC, malignancy confirmed: Original tumour 10-20 mm diameter	31288	0	0	0
Removal of BCC or SCC, malignancy confirmed: >20 mm diameter	31290	30	226	6,782
Removal of residual BCC or SCC, by original GP, specimen sent to histology: Original tumour >20 mm diameter	31291	1	201	201
Removal of residual BCC or SCC, by non-original GP: Original tumour >20 mm diameter	31292	2	355	710
Removal of recurrent BCC or SCC, malignancy confirmed: Original tumour > 20 mm diameter	31293	0	0	0
Removal of recurrent BCC or SCC, by non-original GP, malignancy confirmed: Tumour size unspecified.	31295	6	304	1,822
**Totals**		**4247**		**459,664**

Note:

(i) KC < 10 mm (31255, 31256, 31258, 31265, 31266, 31267, 321280, 31281).

(ii) KC > 20 mm (31275, 31277, 31290).

iii) KC 10-20 mm (31270, 31285, 31287).

(iv) KC > 10 mm (31260, 31261, 31262) [Removed from nose, eyelid, lip, ear, digit or genitalia].

(v) Unspecified size (30071, 31096, 30202, 30203).

Abbreviations: Basal Cell Carcinoma (BCC), Squamous Cell Carcinoma (SCC), General Practitioner (GP).

### Theoretical model

Conceptually an individual with KC could incur three categories of medical costs related to the treatment of KC, two categories of direct treatment costs and one category for related costs. Category 1 costs refer to those procedures, which explicitly identify the treatment of a KC (i.e. the 42 MBS codes detailed above). Category 2 costs refer to non-specific medical treatments, which could also apply to the overall clinical management of KC, for example histopathology, or treatment with antibiotics. Thus, the total cost of treating KC is given by the sum of Category 1 and 2 costs. Category 3 costs refer to the treatment of comorbidities correlated with incidence of KC (e.g. melanoma).

The existence of correlated diseases that generate Category 3 costs could occur because of physiological, environmental, or psychosocial processes. KC is known to be correlated with other cancers [13,22]. Environmental factors such as ultraviolet (UV) radiation are positively correlated with melanoma [23,24] and KC [25]. Negative correlations may also exist, since UV radiation is responsible for the synthesis of vitamin D. Nowson et al. [26] have stated that Vitamin D deficiency is correlated with several diseases including heart disease [27], breast and colon cancer [28], autoimmune diseases such as multiple sclerosis [29], osteoporosis [30,31] and depression [32]. Other implicated diseases include Parkinson’s disease [33], tuberculosis [34] and infectious diseases [31]. Psychosocial factors could affect an individual’s capacity to implement disease prevention measures. Individuals who ignore public health campaigns to mitigate KC might also disregard other disease prevention initiatives.

### Empirical model

To control for the potentially confounding effects of the cost of treating correlated comorbidities the following empirical model was estimated.

$$ C=f\left(KC,\mathbf{R}\mathbf{x},\mathbf{H}\mathbf{x},G,\ A\right) $$

Where

$$ \begin{array}{l}\hfill \\ {}\hfill \\ {}C = \left({\mathrm{MBS}}_{\mathrm{Subsidy}} + {\mathrm{MBS}}_{\mathrm{Co}-\mathrm{payment}}\right) + \left({\mathrm{PBS}}_{\mathrm{Subsidy}} + {\mathrm{PBS}}_{\mathrm{Co}-\mathrm{payment}}\right)\hfill \\ {}\begin{array}{l}KC = 1\ \mathrm{if}\ \mathrm{one}\ \mathrm{of}\ 42\ \mathrm{Category}\ 1\ \mathrm{treatments}\\ {}\mathbf{R}\mathbf{x}=16\ \mathrm{concurrent}\ \mathrm{treatments}\\ {}\mathbf{H}\mathbf{x}=16\;\mathrm{past}\ \mathrm{treatments}\end{array}\hfill \\ {}G=\mathrm{Gender}\hfill \\ {}A=\mathrm{Age}\hfill \end{array} $$

The dependent variable *Cost*, measured in 2012 Australian dollars, was the sum of all MBS and PBS government subsidies and patient co-payments for services utilised from July 2011 to June 2012. Our explanatory variable of interest *KC* was a dichotomous variable equal to one if the participant received one of 42 identified treatments listed in Table 1.

The principal requirement of our empirical model was that it controlled for the cost of concurrent treatments. The vector **Rx,** which contained 16 dichotomous variables indicating the treatment of; three autoimmune diseases (asthma, rheumatoid arthritis and multiple sclerosis), two mental illnesses (depression and anxiety), cardiovascular disease and two associated risk factors (hypertension and hyperlipidaemia), four cancers (melanoma, breast, prostate and colorectal), osteoporosis, Parkinson’s disease, tuberculosis and bronchitis, was constructed from item codes supplied by Medicare Australia.

The Merck Manual [35] was reviewed to formulate a complete list of all diagnostic, medical, surgical and pharmacological treatments used to managed each comorbidity of interest. A search of the MBS [36] and PBS [37] websites was conducted to match each identified treatment to its corresponding item codes. These codes were then used to write the identification algorithms. While not all treatments could uniquely identify a diagnosis, many treatments do indicate a diagnosis. For example, antihypertensive medications are indicative of treatment for hypertension and antidepressants are indicative of treatment for depression. We exercised our clinical judgement to ensure that all included item codes could identify a clinical diagnosis. Nonspecific treatments were removed from the algorithms. For example, morphine, which is sometimes used to treat angina, was not used as an indicator of cardiovascular disease. An itemised list of the 1500 MBS and PBS item codes used to identify the 16 comorbidities are attached in Additional file 1. Comorbidity frequencies are reported in the appendix.

The QSkin survey collected information on co-morbidities as free text. The respondents could report up to two medical conditions that required treatment from a specialist doctor and two cancers (other than skin cancer). The written responses were analysed and used to generate **Hx,** a vector of 16 dichotomous variables indicating previous treatment for the aforementioned diagnoses. The vectors **Rx** and **Hx** are complementary. The former is derived from administrative data and reflects concurrent medical treatment, while the latter is derived from self-reported data and captures prior medical treatment. The costs of treating individuals who report a medical history *vis-à-vis* those who do not, are likely to be systematically different. Therefore, the vector **Hx** was included to capture severity of disease.

A residual treatment category, *treated for other disease*, was created to indicate if the respondent was treated for any other disease. Thus, the reference group for our empirical model were the 51 (1.35%) individuals who incurred no medical or pharmacological costs. Two demographic controls for *gender* and *age* were included in the specification of the empirical model. The coefficient on KC reflects the annual cost to society of 12 months of KC treatment.

Regression using ordinary least squares (OLS) with skewed cost data can result in heteroskedastic errors [38] and biased variance estimates, invalidating *t*-statistics and confidence intervals for regression coefficients [39]. Therefore, a generalised linear model (GLM) with the appropriate distributional family was selected using a modified Park test [40]. The advantage of this approach is that a GLM can accommodate heteroskedasticity through selection of the correct distributional family while generating predictions on the cost scale. This approach also enables one to infer the mean cost directly, without the need to retransform OLS estimates obtained with a logged dependent variable [38].

## Results

The average cost of all medical services utilised by the QSkin respondents, adjusted for age and sex, was AU$2,477. Individuals who were treated for KC consumed an average AU$2,971, while those who were not treated for KC consumed AU$1,918. The difference in means was statistically significant (*p* = 0.01). Conceptually, the AU$1,053 differential could be composed of Category 1, 2 and 3 costs. These costs are distilled as follows.

### 3.1 Category 1 costs

A Category 1 cost was defined as one any of 42 MBS items codes, which directly identified a KC treatment (see Table 1). Columns 1 and 2 report the MBS item description and code, respectively. Column 3 reports service frequency. Columns 4 and 5 report the mean and total costs. The 1,992 individuals treated for KC utilised AU$459,664 in Category 1 services. The mean cost per treated individual was AU$231, of which 77.7% was due to the MBS subsidy and 22.3% was due to the co-payment.

### 3.2 Direct costs

Direct costs were estimated using a GLM with a log-link function and Poisson family distribution, as determined by modified Park test [40]. Table 2 reports the GLM coefficients and a set of marginal effects. The direct cost (i.e., Category 1 and 2 services) of 12 months of KC treatment (AU$667 (*p*-value < 0.01)) is the marginal effect of a dichotomous change in KC from zero to one, with covariates held constant at their means. When gender and age were removed, the estimate increased to AU$676 (*p*-value < 0.01) indicating our estimate is robust with respect to these two covariates. In other specifications, dichotomous variables for education (Nil, School, High school, Trade, Certificate and University) and employment (Full-time, Part-time, Home duties, Student, Retired and Other) were included to control for socioeconomic status. However, *F* tests for joint statistical significance were rejected and their inclusion had no material effect on the coefficient for *KC.*

**Table 2:** **Coefficients and marginal effects for a dichotomous change in keratinocyte cancer (KC)**

**Variables**	**Coefficient**	***p*** **-value**	**Marginal effect of KC**	**Covariates held constant for calculation of marginal effects**	**n**
Keratinocyte cancer (=0/1)	0.36	<0.01	666.5	all covariates @ mean	
*Other medical treatment*					
Treated for other disease (=0/1)	−0.27	0.01	556	=1 & residual @ mean	
Treated for melanoma (=0/1)	0.40	<0.01	988	=1 & residual @ mean	53
Treated for hypertension (=0/1)	0.09	0.15	719	=1 & residual @ mean	645
Treated for hyperlipidaemia (=0/1)	0.04	0.55	686	=1 & residual @ mean	1036
Treated for cardiovascular disease (=0/1)	0.56	0.00	953	=1 & residual @ mean	1342
Treated for diabetes (=0/1)	0.16	0.02	774	=1 & residual @ mean	354
Treated for breast cancer (=0/1)	0.41	<0.01	983	=1 & residual @ mean	187
Treated for colorectal cancer (=0/1)	0.36	<0.01	926	=1 & residual @ mean	279
Treated for prostate cancer (=0/1)	0.38	<0.01	952	=1 & residual @ mean	227
Treated for asthma (=0/1)	0.19	<0.01	785	=1 & residual @ mean	507
Treated for rheumatoid arthritis (=0/1)	0.30	<0.01	862	=1 & residual @ mean	546
Treated for multiple sclerosis (=0/1)	0.83	<0.01	1,522	=1 & residual @ mean	10
Treated for depression (=0/1)	0.17	<0.01	771	=1 & residual @ mean	602
Treated for anxiety (=0/1)	0.30	0.01	895	=1 & residual @ mean	118
Treated for osteoarthritis (=0/1)	0.40	<0.01	939	=1 & residual @ mean	527
Treated for Parkinson’s disease (=0/1)	0.23	0.23	840	=1 & residual @ mean	22
Treated for tuberculosis (=0/1)	0.90	< 0.01	1,626	=1 & residual @ mean	49
Treated for bronchitis (=0/1)	−0.01	0.96	663	=1 & residual @ mean	85
*Medical history*					
History of hypertension (=0/1)	0.14	0.18	-	-	-
History of hyperlipidaemia (=0/1)	0.21	0.39	-	-	-
History of cardiovascular disease (=0/1)	0.01	0.85	-	-	-
History of diabetes (=0/1)	0.07	0.46	-	-	-
History of breast cancer (=0/1)	0.21	0.06	-	-	-
History of colorectal cancer (=0/1)	−0.29	0.03	-	-	-
History of prostate cancer (=0/1)	0.08	0.60	-	-	-
History of asthma (=0/1)	0.27	0.22	-	-	-
History of rheumatoid arthritis (=0/1)	0.46	0.02	-	-	-
History of multiple sclerosis (=0/1)	1.65	< 0.01	-	-	-
History of depression (=0/1)	0.04	0.75	-	-	-
History of anxiety (=0/1)	0.40	0.04	-	-	-
History of osteoarthritis (=0/1)	−0.14	0.39	-	-	-
History of Parkinson’s disease (=0/1)	0.51	0.11	-	-	-
History of tuberculosis (=0/1)	−0.15	0.62	-	-	-
History of bronchitis (=0/1)	−1.03	< 0.01	-	-	-.
*Other variables*					
Female (=0/1)	−0.06	0.29	-	-	-
Age (years)	0.00	0.13	-	-	-.
Constant	6.67	< 0.01	-	-	-.

Note: All reported marginal effects for keratinocyte cancer were statistically significant (*p*-value < 0.05).

The marginal effects of KC were also estimated controlling for comorbidity and age. Table 2, Column 4 reports the marginal effect of a dichotomous change in KC with each comorbidity set equal to one, and all other covariates held constant at their mean. Hence, for individuals treated for melanoma (n = 53), the marginal effect of 12 months of KC treatment was AU$988. The marginal effect of KC was estimated for each age, 40 through to 70. At age 40, the marginal effect of 12 months of KC treatment was AU$614. The marginal effect increased linearly, by AU$3.30 per year, to AU$713 at age 70.

### Cost summary

The results presented in Table 3, Column 2 summarise the principal findings of this paper. The annual MBS subsidy per KC treatment was AU$677 per individual. As this estimate is a derived value, we cannot directly differentiate between the subsidy and co-payment. If the cost distribution was comparable to Category 1 services, this would imply the MBS subsidy was AU$518 (77.7%) and the co-payment AU$149 co-payment (22.3%). The average cost of Category 1 services was AU$230 (see Table 1 for description costs). The cost of Category 2 services used to treat KC was AU$437 (i.e. AU$667 – AU$230). A further AU$386 (i.e. AU$1,053 – AU$667) was spent on Category 3 services treating diseases correlated with KC.

**Table 3:** **Costs of medical care used utilised by individuals with KC**

	**Estimated mean costs**		**Observed total costs**	
**Costs categories**	**Costs (AU$) [95% CI]**	**Method**	**Costs (AU$)**	**Source**
Category (1)	231 [217 to 244]	${\displaystyle \sum_{i=1}^k} Cat1/n$	459,664	See Table 1
Category (2)	436 [240 to 632]*	Cat. (1 + 2) - Cat. 1	643,993	See Table 4
Category (1 + 2)	667 [470 to 863]	GLM		
Category (3)	386 [47 to 725]*	Correlated with KC - Cat. (1 + 2)		
Correlated with KC	1,053 [776 to 1,330]	${\displaystyle \sum_{i=1}^n} Cost/n-{\displaystyle \sum_{i=m}^m} Cost/m$		
KC = 0	1,918 [1,745 to 2,091]	${\displaystyle \sum_{i=1}^m} Cost/m$		
KC = 1	2,971 [2,760 to 3,182]	${\displaystyle \sum_{i=1}^n} Cost/n$		

Note:

*n* = Number with KC.

*m* = Number without KC.

*k* = Number of Category 1 services.

*The 95% CI for the difference between two means (*ā* and $\overline{b}$) was estimated by $\pm 1.96*\sqrt{SE\frac{2}{a}+SE\frac{2}{b}}$ [41].

### Category 2 costs

When estimated with OLS the errors were not normally distributed (Shapiro-Wilk test: *W* = 0.48 (*p* < 0.01)) and heteroskedastic (*χ* ^2^ (1) = 4148.8 (*p*-value < 0.01)). We estimate our model using a GLM. Category 2 costs account for 66% of the costs attributable to the management of KC. Thus our best estimate of total Category 2 costs related to the treatment of KC is AU$868,512 (i.e. 1,992 * AU$436). Due to their magnitude, we sought to identify those Category 2 costs in the following way. First, the data were transformed into wide format, such that each observation was now a medical service. All Category 1 services were removed. The frequencies of the residual services were cross-tabulated with a dichotomous variable equal to one if the service was delivered to an individual with KC and zero if otherwise. The frequency difference, estimates the number of Category 2 and 3 services utilised.

$$ \mathrm{Freq}{.}_{\mathrm{KC}=1}\hbox{--}\ \mathrm{Freq}{.}_{\mathrm{KC}=0} = \mathrm{Category}\ 2\ \mathrm{services} + \mathrm{Category}\ 3\ \mathrm{services}. $$

Table 4 presents a summary of our findings. Column 1 lists clinical services groups, with their MBS item codes listed in the table notes. Columns 2 and 3 list the treatment frequencies for the cohorts with and without a KC and Column 4 reports the frequency differences. The cost of each service category is given by the product of Columns 4 and 5 and is reported in Column 6. After inspecting the service descriptors, we could identify three groups of medical services, which could plausibly be attributed to the treatment of KC. In our study, the KC cohort consumed an additional AU$191,115 on reconstructive surgeries, AU$167,096 on pathology and AU$453,623 on consultation fees.

**Table 4:** **Potential category 2 costs**

	**Frequencies**				
**MBS schedule service description**	**KC = 1**	**KC = 0**	**Difference**	**Mean cost (AU$)**	**Total cost (AU$)**
***Reconstructive surgeries***					
Single stage local flap	167	9	158	404	63,832
Free grafting	72	7	65	552	35,880
Lip, eyelid or ear, full thickness wedge	17	1	16	393	6,288
H-Flap or double advancement flap	16	1	15	351	5,265
Vermilionectomy	5	1	4	511	2,044
Whole thickness reconstruction of eyelid	1	0	1	846	846
Tumor, cyst, ulcer or scar removal	510	139	371	107	39,697
Lens replacement	30	23	7	1553	10,871
Premalignant skin Lesions	678	170	508	32	16,256
Neoplastic skin lesions	244	63	181	56	10,136
***Pathology***					
Histology	1915	332	1583	104	164,632
Microscopy and culture of skin	110	33	77	32	2,464
***Medical fees***					
Medical fees	6685	4234	2451	60	147,060
Initial or subsequent visit to specialist	4111	2250	1861	104	193,544
Anaesthetic for procedures on skin	102	19	83	300	24,900
Pre anaesthetic consult	491	319	172	336	57,723
Radiation Oncology	337	133	204	149	30,396

Note: Service definitions: MBS item numbers.

Medical attendance fees: 3, 23, 36, 44, 193, 197, 199, 2501, 2503, 2504, 2517, 2521, 2525, 2546, 2552, 5000, 5020, 5040, 5060, 86014.

Initial or subsequent visit to specialist: 104, 105, 110, 116.

Anaesthetic procedures on skin: 20100, 20160, 20300, 20400, 20420, 20700, 20800, 20820, 20900, 21110, 21460, 21600, 21800.

Pre-Anaesthetic consults: 17640, 17645, 17650, 17655, 17680, 17610, 17615, 17620, 17625, 17640, 17645, 17650, 17655, and 17680.

Radiation Oncology: 15269, 15248, 15251, 15257, 15260, 15263, 15266, 15272, 15705.

Single stage local flap: 45200, 45203, 45206, 45000, 45003.

Free grafting: 45400, 45403, 45439, 45442, 45445, 45448, and 45451.

Lip, eyelid or ear, full thickness wedge: 45665.

H-flap or double advancement flap: 45207.

Vermilionectomy: 45668, 45669.

Whole thickness reconstruction of eyelid: 45614.

Tumour, cyst, ulcer or scar removal: 31200, 31205, 31210, 31215, 31220, 31225, 31230, 31235, 31240, 52045.

Lenses replacement: 42702.

Premalignant skin lesions: 30192.

Neoplastic skin lesion: 30195.

Histology: 72816, 72817, 72818, 72823, 72824, 72825, 72826, 72827, 72828, 72830, 72836, 72838.

Microscopy and culture of skin: 69306.

Abbreviations: Medical Benefits Schedule (MBS), Keratinocyte Cancer (KC).

**Figure 1:**
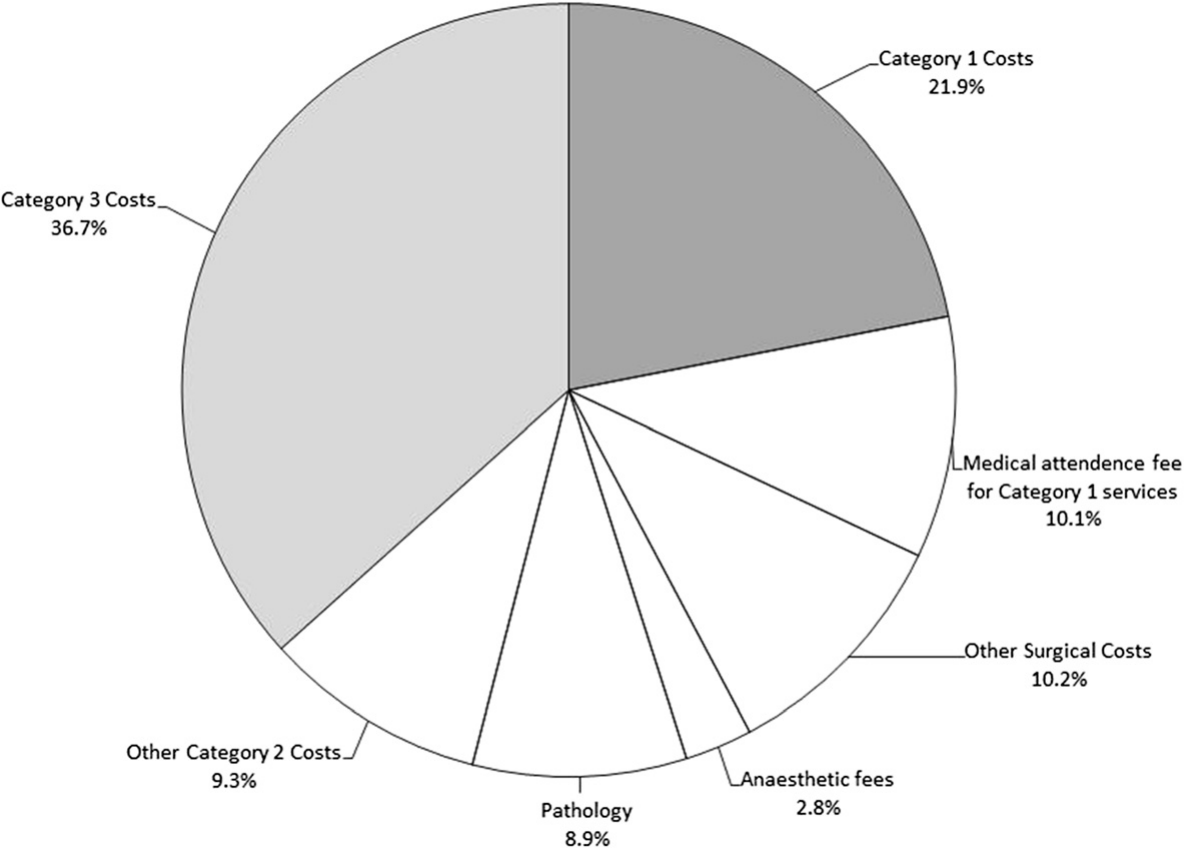
**The costs of treating individuals with KC (Total cost AU$1,053).**

The data presented in Figure 1, summarise the analyses presented in the paper. In our sample, the total cost of all additional medical services consumed by people with a KC is AU$1,053 per year. The costs of direct treatments or Category 1 costs account for 22% of total costs. A detailed enumeration of these costs is reported in Table 1. Other generic treatments of KC or Category 2 costs account for 41% of the total. The principal cost components are approximately medical attendance fees 10.1%, other surgical costs 10.2%, anaesthetic fees 2.8% and pathology 8.9%. Other unspecified services contribute a further 9.3% to total costs. The cost of correlated comorbidities accounts for 37% of the total cost.

## Discussion

The medical record is comprised of a complex array of clinical data, which if available, can enhance the quality of analysis in health research. For this work, we utilised an administrative dataset to reconstruct the medical record of a sample of study participants from first principals. Our motivation was to estimate and identify the direct and associated costs of treating KC. Including controls for the medical record enabled the cost of KC treatment to be estimated. We identified 16 comorbidities of interest. Each comorbidity was identified by a list of diagnostic, medical, surgical and pharmacological item codes associated with the treatment of that disease. Multivariate regression was used to generate an estimate of cost, which controlled for the patient’s medical record with 16 dichotomous covariates.

The decision to identify three categories of medical costs was motivated by our desire to provide a more comprehensive description of the medical treatments utilised by patients with KC, than is currently available in the literature. Whereas Fransen et al. utilised a minimalist definition of KC, where a “treatment” was defined by 37 Category 1procedures directly associated with KC excision, our method has included Category 2 procedures in the analysis. Results derived from our GLM suggest that 12 months of KC treatment cost AU$667 per patient. Given a national age-standardised rate (ASR) of 3,271 KC services per 100,000 people [7], the implied cost of MBS services to the nation is AU$228 million per year^b^, is considerably higher than AU$93.5 million reported by Fransen et al. [7].

Category 2 costs were found to account for 65% (AU$436) of the total KC treatment (AU$667). The robustness of this result was tested by constructing an *ad hoc* tabulation of medical services we could attribute to the treatment of KC. In addition to the Category 1 services, we could identify three distinct sub-groups of Category 2 costs, which were utilised by the KC cohort. Firstly, AU$191,000 was spent on reconstructive surgeries. Localised disfigurement is often a consequence of KC. Secondly, an additional AU$167,000 was spent on *pathology.* Histological examination of excised tissue plays a pivotal role in differentiating benign and malignant KC and therefore we believe that a significant proportion of these costs were due to KC. Thirdly the KC cohort consumed an additional AU$453,623 in medical fees.

While not all medical fees can be attributed to the treatment of KC, a pro rata adjustment implied by our GLM model, suggests that at least 63% [i.e. (667/1053)*100] of these medical fees were incurred managing KC. The surgical management of KC *vis-à-vis*c correlated comorbidities is likely to be labour intensive as surgical management in the ambulatory setting can require separate appointments to diagnose biopsy, treat and provide follow-up care, unlike medical conditions, which can be diagnosed and treated within a single appointment. Inspection of the data suggests that in excess of AU$644,000 (74%) of the Category 2 costs implied by the GLM estimate can readily be identified^c^.

A strength of this study was our utilisation of data from a matched population-based sample of individuals linked to an administrative cost dataset supplied by the Department of Health, Australian Government. Our decision to control for medical history was justified because the distribution of comorbid disease was not randomly distributed amongst cases and controls. Treatment rates for hypertension (18.5% vs. 15.9%), colorectal cancer (8.5% vs. 6.1%) and prostate cancer (6.9% vs. 5.1%) were higher for individuals treated for KC. As a result, a large proportion of the comorbities were statistically significant. For individuals who received treatment for one of the 16 comorbidities the marginal effect of a KC was greater than the mean (AU$667). Patients with other cancer diagnoses (e.g. melanoma, breast, colorectal and prostate) had KC costs about AU$300 (45%) higher than those without. Furthermore, each additional year of life increased the marginal cost of KC treatment by AU$3.30 per year.

A recently published paper by Ong et al. [42] analysed the ICD-10 codes included in 8 million medical records from the United Kingdom. In patients aged 45–69 years, they report the relative risks of being treated for melanoma (9.4), breast cancer (1.25), prostate cancer (1.21), colon cancer (1.30) and rectal cancer (2.59) given a diagnosis of KC, which closely correspond to our estimates for melanoma (14.73), breast cancer (1.24), prostate cancer (1.37) and colorectal cancer (1.38)^d^. This suggests that the analysis of medical invoices can offer an accurate insight into the patients’ medical history.

Our study has a number of limitations. While our study collected administrative data that is likely to be comprehensive in most medical services for KC, other omitted costs to the health system will underestimate our figures. We did not have access to linked data for non-Medicare services, the inpatient costs for a hospital admission or other community services. Nor did our survey data include out-of-pocket expenses or indirect costs related to interruptions to employment associated with treatments. Furthermore, the sample includes individuals from one state of Australia with very high rates of skin cancer and preventive behaviour may differ from elsewhere. In Queensland, the per patient costs for treating KC may be minimised through the predominance of care delivered in office-based GP clinics and dedicated GP-run skin cancer clinics. The principal methodological limitation of our approach remains the exclusion of other relevant comorbidities from our statistical models. If relevant, the non-inclusion of these variables is likely to result in an over estimation of the Category 2 costs associated with treating KC and an underestimation of the Category 3 costs of comorbidities correlated with KC.

## Conclusion

The methodology used in this paper has a much broader application than estimating the costs of KC. Being able to control for confounding effects within an individual’s medical history can enhance empirical analysis in healthcare. In this paper, medial service codes were utilised to reconstruct a medical record from first principles. These data have greatly enriched the breadth of our analysis, thus enabling further examination and reporting of a much richer description of the determinants of cost.

## Endnotes

^a^Our list of 42 MBS codes contained all of the 37 codes identified by Fransen et al. [7].

^b^-Age Standardised Rate (ASR) of KC services per 100,000 in 2011 = 3,271 [6].

- Population = 22,319,006 [6].

- MBS subsidy per 12 month KSC treatment = AU$518 (i.e. 667 * 0.777) (QSkin).

- KSC treatments per year = 1.66 (QSkin).

- ASR/100,000 * Population* MBS subsidy/ KSC treatments per year = AU$228 m.

^c^AU$191,115 + AU$167,096 + (0.63 * AU$453,623) = AU$643,933.

^d^A complete set of estimated relative risks for each comorbidity are available from the corresponding author upon request.

## Additional file

Additional file 1:
**The Medicare Benefits Scheme item codes used to identify the covariates used to reconstruct the a medical history.**
