# Supplementary material for: A reconstruction of a medical history from administrative data: with an application to the cost of skin cancer
Source: Health Econ Rev. 2015 Feb 11;5:4. doi: 10.1186/s13561-015-0042-x (PMC4384899; doi:10.1186/s13561-015-0042-x)
Supplement: Additional file 1: — The Medicare Benefits Scheme item codes used to identify the covariates used to reconstruct the a medical history. [file 13561_2015_42_MOESM1_ESM.docx]

Additional file 1

The Medicare Benefits Scheme item codes used to identify the covariates used

To Reconstruct the a Medical History

|  |
| --- |
| **KC:** |
| Diagnostic Tests: nil |
| Medical & Surgical Treatments: MBS items30196, 30197, 30202, 30203, 31255, 31256, 31257, 31258, 31260, 31261, 31262, 31263, 31265, 31266, 31267, 31268, 31270, 31271, 31272, 31273, 31275, 31276, 31277, 31278, 31280, 31281, 31282, 31283, 31285, 31286, 31287, 31288, 31290, 31291, 31292, 31293, 31295 |
| Pharmacology**:** nil |
| **MELANOMA:** |
| Diagnostic Tests: nil |
| Medical & Surgical Treatments: MBS items31300, 31305, 31310, 31315, 31320, 31325, 31330, 31335, 52042 |
| Pharmacology**:** 4437M, 7245H |
| **HYPERTENSION:** |
| Diagnostic Tests: nil |
| Medical & Surgical Treatments: nil |
| Pharmacology: PBS items1585K, 5285E, 5286F, 5287G, 5288H, 5289J, 8504N, 9314F, 9315G, 8477E, 8624X, 8400D, 8401E, 1484D, 1486F, 1280J, 2136K, 8404H, 8405J, 2161R, 2166B, 2170F, 8589C, 8590D, 9372G, 9373H, 9374J, 9481B, 9482C, 2436F, 8532C, 2190G, 2845R, 8449Q, 1486F, 8879H, 8880J, 2339D, 2340E, 1280J, 1081X, 2243C, 2811Y, 2825Q, 5543R, 5544T, 8604W, 9311C, 9312D, 9316H, 1324Q, 8732N, 8733P, 8734Q, 8735R, 3062E, 3065H, 8255L, 8256M, 8257N, 8258P, 1566K, 1567L, 2565B, 2566C, 2899N, 2043M, 8398B, 1081X, 2243C, 1459T, 1515R, 1516T, 3475X, 5108W, 8010N, 8011P, 8026K, 8027L, 8028M, 8119H, 8171C, 1312C, 1313D, 1335G, 8480H, 2857J, 9387C, 1241H, 1248Q, 1250T, 1254B, 2206D, 2207E, 2208F, 2751T, 2752W, 9049G, 9050H, 9051J, 9052K, 9053L, 9054M, 9055N, 9056P, 9346X, 9347Y, 9348B, 9349C, 5459H, 5460J, 9375K, 9376L, 9377M, 5285E, 5286F, 5287G, 5288H, 5289J, 5292M, 5293N, 5294P, 8978M, 8979N, 8980P, 8981Q, 2361G, 2366M, 2367N, 2626F, 2629J, 1147J, 1148K, 1149L, 8760C, 2456G, 2457H, 2458J, 1182F, 1183G, 8400D, 8401E, 1316G, 1944H, 1945J, 1946K, 8470T, 8668F, 9120B, 9121C, 9122D, 2626F, 2629J, 1968N, 1969P, 1970Q, 8589C, 8590D, 1368B, 1369C, 1370D, 8477E, 9144G, 9145H, 3050M, 3051N, 8704D, 9006B, 9007C, 9008D, 9346X, 9347Y, 9348B, 9349C, 2190G, 2845R, 8449Q, 2791X, 2792Y, 2793B, 8758Y, 2857J, 9387C, 3141H, 3145M, 1629R, 1478T, 1479W, 1480X, 4396J, 4397K, 4398L, 4399M, 1639G, 1640H, 2313R |
| **LIPIDEMIA:** |
| Diagnostic Tests: MBS items 66500, 66536 |
| Medical & Surgical Treatments: nil |
| Pharmacology: PBS items 8213G, 8214H, 8215J, 8521L, 9230T, 9231W, 9232X, 9233Y, 2863Q, 8023G, 9234B, 9234B, 9235C, 9236D, 2833D, 2834E, 8197K, 8829Q, 9237E, 9238F, 9239G, 9240H, 3402C, 3403D, 3404E, 3405F, 9043Y, 9045C, 2011W, 2012X, 2013Y, 8173E, 8313M, 9241J, 9242K, 9243L, 9244M, 9245N, 8881K, 8882L, 9483D, 9484E, 8757X, 2967E, 9249T, 1224K, 9250W |
| **CARDIOVASCULAR DISEASE:** |
| Diagnostic Tests: MBS items11700, 11701, 11702, 11708, 11709, 11710, 11711, 11712, 11713, 66506, 66518, 66519, 55113, 55114, 55115, 55119, 55120, 55130, 55131, 55135, 55136, 57360, 57361, 61313, 61316, 61317, 61320, 61656, 61657, 61658, 61659, 61660, 59903, 59912 |
| Medical & Surgical Treatments: MBS items 38200, 38218, 38275, 22075, 38209, 38212, 38213, 38456, 38209, 38212, 38350, 38353, 38356, 38365, 38368, 38371, 38654, 13400, 38640, 22060, 38285, 38365, 38368, 38654, 38358, 38470, 38473, 38475, 38477, 38478, 38480, 38481, 38483, 38485, 38487, 38488, 38489, 38490, 38493, 38496, 38497, 38498, 38500, 38501, 38503, 38504, 38505, 38506, 38507, 38508, 38509, 38512, 38515, 38518, 38550, 38553, 38556, 38559, 38562, 38565, 38568, 38571, 38572, 38577, 38588, 38600, 38603, 38609, 38612, 38613, 38615, 38618, 38621, 38624, 38627, 38637, 38640, 38643, 38647, 38650, 38653, 38654, 38656, 38670, 38673, 38677, 38700, 38703, 38706, 38709, 38712, 38715, 38718, 38721, 38724, 38727, 38733, 38736, 38739, 38742, 38745, 38748, 38751, 38754, 38757, 38760, 38763, 38766, 21941, 13818, 13876, 21941, 21942, 22012, 22014, 22015, 38203, 38206, 38209, 38212, 38213, 38270, 38275, 38285, 38350, 38353, 38356, 38358, 38365, 38368, 38371, 38384, 38387, 38654, 38670, 38673 |
| Pharmacology: PBS items1010E, 4076M, 4077N, 4078P, 5018D, 8202Q, 4179Y, 5436D, 9296G, 9495R, 9496T, 1076P, 1463B, 1466E, 5434B, 5435C, 8262W, 8263X, 8264Y, 8510X, 8558K, 8639Q, 8640R, 8716R, 9195Y, 9196B, 1229Q, 1296F, 2816F, 5445N, 8269F, 8271H, 8603T, 8641T, 8642W, 8643X, 8956J, 8957K, 8958L, 8959M, 8960N, 1081X, 2243C, 2811Y, 2825Q, 5543R, 5544T, 8604W, 9311C, 9312D, 9316H, 1324Q, 8732N, 8733P, 8734Q, 8735R, 3062E, 3065H, 8255L, 8256M, 8257N, 8258P, 1566K, 1567L, 2565B, 2566C, 2899N, 2043M, 8398B, 1081X, 2243C, 1459T, 1515R, 1516T, 3475X, 5108W, 8010N, 8011P, 8026K, 8027L, 8028M, 8119H, 8171C, 8213G, 8214H, 8215J, 8521L, 9230T, 9231W, 9232X, 9233Y, 2863Q, 8023G, 9234B, 9234B, 9235C, 9236D, 2833D, 2834E, 8197K, 8829Q, 9237E, 9238F, 9239G, 9240H, 3402C, 3403D, 3404E, 3405F, 9043Y, 9045C, 2011W, 2012X, 2013Y, 8173E, 8313M, 9241J, 9242K, 9243L, 9244M, 9245N, 8881K, 8882L, 9483D, 9484E, 1147J, 1148K, 1149L, 8760C, 2456G, 2457H, 2458J, 1182F, 1183G, 8400D, 8401E, 1316G, 1944H, 1945J, 1946K, 8470T, 8668F, 9120B, 9121C, 9122D, 2626F, 2629J, 1968N, 1969P, 1970Q, 8589C, 8590D, 1368B, 1369C, 1370D, 8477E, 9144G, 9145H, 3050M, 3051N, 8704D, 9006B, 9007C, 9008D, 9346X, 9347Y, 9348B, 9349C, 2190G, 2845R, 8449Q, 2791X, 2792Y, 2793B, 8758Y, 2857J, 9387C |
| **DIABETES:** |
| Diagnostic Tests: MBS items66542, 66551, 66554, 66557, 701, 703, 705, 707 |
| Medical & Surgical Treatments: MBS items2517, 2518, 2521, 2522, 2525, 2526, 2620, 2622, 2624, 2631, 2633, 2635, 81100, 81105, 81110, 81115, 81120, 81125, 81305 |
| Pharmacology: PBS items 8435Y, 8571D, 8609D, 9040T, 9039R, 1921D, 9224L, 1711C, 1533Q, 1761Q, 1426C, 1763T, 2062M, 8084L, 8212F, 8390N, 8874C, 1713E, 1531N, 1762R, 1801T, 2430X, 3439B, 8607B, 9435N, 2440K, 2449X, 8535F, 9302N, 2939Q, 8450R, 8451T, 8452W, 8533D, 9449H, 9450J, 9451K, 9059T, 9060W, 9061X, 9062Y, 5474D, 5475E, 5476F, 8810Q, 8811R, 8838E, 8188Y, 8189B, 8689H, 8690J, 8694N, 8695P, 8696Q, 3387G, 8983T, 9180E, 9181F, 9182G, 3415R, 3423E, 3424F |
| **BREAST CANCER:** |
| Diagnostic Tests: MBS items59300, 59301, 59303, 59304, 59306, 59309, 59312, 59318, 59319, 63464, 63467 |
| Medical & Surgical Treatments: MBS items 20404, 30299, 30300, 30302, 30303, 31500, 31503, 31506, 31509, 31512, 31515, 31518, 31521, 31524, 31527, 31530, 31533, 31536, 31542, 31548, 31551, 31554, 45524, 45527, 45530, 45533, 45536, 45539, 45542, 45548, 45551, 45552, 45553, 45554, 13015, 15236, 15266, 15251, 15266 |
| Pharmacology: 1880Y, 2109B, 2110C |
| **COLORECTAL CANCER:** |
| Diagnostic Tests: MBS items66650, 66764, 73809, 73836, 56552, 56554, 57360, 57361, 11820, 21927, 58912, 58916, 58917, 58920 |
| Medical & Surgical Treatments: MBS items 20841, 20844, 32004, 32005, 32009, 32012, 32015, 32018, 32021, 32051, 32054, 32057, 32000, 32003, 32004, 32006, 32024, 32025, 32026, 32028, 32030, 32033, 32039, 32042, 32045, 32046, 32060, 32063, 32066, 32069, 30382, 30562, 32220, 32221, 30680, 30682, 30684, 30686, 32084, 32087, 32090 |
| Pharmacology: nil |
| **PROSTATE CANCER:** |
| Diagnostic Tests: MBS items66656, 66660 |
| Medical & Surgical Treatments: MBS items15218, 15338, 37201, 37202, 37207, 37208, 37209, 37210, 37211, 37212, 37215, 37217, 37218, 37219, 37220, 37224, 37230, 37233, 37203, 37206, 37200, 37210, 37211 |
| Pharmacology: nil |
| **ASTHMA:** |
| Diagnostic Tests: MBS items 11503, 11506, 11509, 11512 |
| Medical & Surgical Treatments: nil |
| Pharmacology: PBS items1299J, 1103C, 2000G, 2001H, 2003K, 3495Y, 3496B, 3497C, 8288F, 8354Q, 8136F, 8239P, 8240Q, 8430Q, 8431R, 8432T, 8517G, 8518H, 8519J, 8141L, 1542E, 4089F, 4090G, 8238N, 8671J, 8626B, 8406K, 8407L, 8408M, 8409N, 2065Q, 2066R, 2070Y, 2071B, 2072C, 4092J, 8625Y, 8750M, 8796Y, 8147T, 8148W, 8149X, 8346G, 8516F, 8627C, 8628D, 2614N, 2634P, 8230E, 8231F, 9745X, 9746Y |
| **RHEUMATOID ARTHRITIS:** |
| Diagnostic Tests: MBS items71106, 12315, 12318 |
| Medical & Surgical Treatments: nil |
| Pharmacology: PBS items1299J, 1300K, 1302M, 4046Y, 5076E, 5077F, 5079H, 5361E, 5362F, 5363G, 5364H, 5365J, 5366K, 4190M, 5514F, 8699W, 3190X, 3192B, 5123P, 5124Q, 5368M, 5370P, 2454E, 2757D, 5126T, 5128X, 5377B, 5378C, 5379D, 5380E, 1895R, 1896T, 1897W, 1898X, 5201R, 5202T, 5203W, 5204X, 8439E, 8440F, 8561N, 8562P, 8887R, 8888T, 1512N, 5449T, 5450W, 8373Q, 8374R, 8375T, 1622J, 1623K, 1623K, 1818Q, 2272N, 2395C, 2396D, 4502Y, 4512L, 7250N, 7251P, 2093E, 2096H, 9208P, 9209Q, 1928L, 2981X, 5148Y, 5263B, 5264C, 8054X, 8055Y, 8128T, 8618N, 8834Y, 2117K, 2118L, 2990J, 5233K, 1934T, 1935W, 1936X, 2687K, 2688L, 1266P, 4327R, 7226H, 1220F, 1221G, 5605B, 9621J, 4613T, 4614W, 4615X, 7257Y, 7258B, 7259C, 9544H, 9611W, 5281Y, 5282B, 5283C, 5284D, 8737W, 8741C, 8961P, 8962Q, 8963R, 8964T, 8965W, 8966X, 9033K, 9034L, 9077R, 9078T, 9099X, 9100Y, 9101B, 9102C, 9103D, 9104E, 9186L, 9187M, 9188N, 9189P, 9190Q, 9191R, 9425C, 9426D, 9427E, 9428F, 9661L, 9662M, 9663N, 9678J, 9679K, 9680L, 1954W, 1963H, 1964J, 3445H, 3446J, 3447K, 3448L, 3449M, 3450N, 5733R, 5734T, 5735W, 6367D, 8637N, 8638P, 8778B, 8779C, 9035M, 9036N, 9037P, 9085E, 9086F, 9087G, 9088H, 9089J, 9090K, 9091L, 9429G, 4284L, 5753T, 5753T, 5754W, 5754W, 5755X, 5756Y, 5757B, 5758C, 6397Q, 6448J, 6496X, 9612X, 9613Y, 9617E, 9654D |
| **MULTIPLE SCLEROSIS:** |
| Diagnostic Tests: MBS items63507, 63508, 63510, 63511, 56047, 66756, 69321, 71059, 71062, 71139, 71143, 71145 |
| Medical & Surgical Treatments: MBS items14227, 14230, 14233, 14236, 14239, 14242 |
| Pharmacology: PBS items2729P, 2730Q, 5617P, 6284R, 1834M, 1835N, 4591P, 4592Q, 4593R, 4594T, 4595W, 8389M, 8505P, 8559L |
| **DEPRESSION:** |
| Diagnostic Tests: nil |
| Medical & Surgical Treatments: MBS items80100, 80105, 80110, 80115, 80120, 80125, 80130, 80135, 80140, 80145, 80150, 80155, 80160, 80165, 80170, 2700, 2701, 2712, 2713, 2715, 2717, 855, 857, 858, 861, 864, 866, 300, 302, 304, 306, 308, 310, 312, 314, 316, 318, 2721, 2723, 2725, 2727, 291, 293, 296, 297, 299, 14224, 20104 |
| Pharmacology: PBS items1011F, 1012G, 1013H, 1357K, 1358L, 1434L, 1561E, 1900B, 1973W, 2236Q, 2237R, 2242B, 2417F, 2418G, 2420J, 2421K, 2429W, 2444P, 2522R, 2523T, 2856H, 8003F, 8174F, 8220P, 8270G, 8301X, 8302Y, 8465M, 8512B 8513C, 8700X, 8701Y, 8702B, 8703C, 8710K, 8836C, 8837D, 8849R, 8855C, 8857E, 8868R, 8883M, 9155W, 9156X, 9197C, 9365X, 9432K, 9433L |
| **ANXIETY:** |
| Diagnostic Tests: nil |
| Medical & Surgical Treatments: nil |
| Pharmacology: PBS items2130D, 2131E, 2132F, 8118G, 1805B, 1806C, 1807D, 1808E, 3478C, 5337X, 5338Y, 5339B, 5340C, 5341D, 5342E, 2558P, 3161J, 3162K, 3458B, 5071X, 5072Y, 5073B, 5355W, 5356X, 5357Y, 5358B, 3132W, 3133X, 3134Y, 3135B, 5192G, 5193H, 5371Q, 5372R, 5373T, 5374W, 4150K, 4151L |
| **OSTEOPOROSIS:** |
| Diagnostic Tests: MBS items12306, 12309, 12312, 12315, 12318, 12321, 12323, 66500, 66608, 66695 |
| Medical & Surgical Treatments: nil |
| Pharmacology: PBS items2191H, 4443W, 4444X, 8481J, 8482K, 8621R, 8972F, 9391G, 2220W, 4059P, 8899J, 8973G, 2254P, 2215N, 8090T, 8511Y, 2194L, 2224C, 9012H, 9183H, 2273P, 9351E, 3036T, 5110Y, 5457F, 6371H, 9288W, 9350D, 9653C, 9411H |
| **PARKINSON'S DISEASE:** |
| Diagnostic Tests: nil |
| Medical & Surgical Treatments: MBS items40801, 40850, 40851, 40852, 40854, 40856, 40860, 40862 |
| Pharmacology: PBS items1109J, 1110K, 2544X, 2362H, 3038X, 3457Y, 1242J, 1245M, 1255C, 8970D, 9743T, 9744W, 2225D, 2226E, 2227F, 2228G, 2229H, 2231K, 8218M, 8219N, 8797B, 8798C, 8799D, 9292C, 9344T, 3016R, 5609F, 5610G, 5611H, 9607P, 9640J, 9647R, 1443Y, 1445C, 1446D, 1559C, 8393R, 8394T, 2808T, 2809W, 2810X, 3418X, 3419Y, 3420B, 3421C, 3422D, 5143Q, 5145T, 9151P, 9152Q, 9153R, 9393J, 9394K, 1973W, 1952R, 8367J |
| **TUBERCULOSIS:** |
| Diagnostic Tests: MBS items73811, 69318, 69324, 69327, 69330 |
| Medical & Surgical Treatments: nil |
| Pharmacology: PBS items1554T, 1981G, 1982H, 1983J, 1984K, 8025J, 6195C, 9541E |
| **BRONCHITIS:** |
| Diagnostic Tests: nil |
| Medical & Surgical Treatments: nil |
| Pharmacology: PBS items (i) β-Agonist 1099W, 1103C, 2000G, 2001H, 3495Y, 3496B, 3497C, 8288F (ii) Antibiotics 1884E, 1886G, 1887H, 1888J, 1889K, 3300Q, 3301R, 3302T, 3310F, 3393N, 5225B, 8581P, 8705E, 9714G, 1891M, 1892N, 5006L, 5008N, 5009P, 5011R, 8254K, 8319W, 1800R, 2702F, 2703G, 2707L, 2708M, 2709N, 2711Q, 2714W, 2715X, 3321T, 5082L, 9105F, 9106G, 9107H, 9108J, 4115N, 5616N, 6221K, 8200N, 8201P, 8336R, 2951H, 3103H, 3390K, 3391L |

Note:

1. The medical treatments for each diagnosis were obtained from the Merck Manual([Porter and Kaplan 2011](#_ENREF_26)). The MBS and PBS item codes were sourced from the MBS (<http://www9.health.gov.au/mbs/search.cfm> ) and PBS (<http://www.pbs.gov.au/pbs/home>) websites.
2. The binary variables KC through to Tuberculosis were =1 if the individual had **any** of the identified MBS or PBS item codes. The binary variable Bronchitis was =1 if the individual was treated with (i) β-Agonist inhaler **and** (ii)an antibiotic.
